# Comparing Transcriptome Profiles of Saccharomyces Cerevisiae Cells Exposed to Cadmium Selenide/Zinc Sulfide and Indium Phosphide/Zinc Sulfide

**DOI:** 10.3390/genes12030428

**Published:** 2021-03-17

**Authors:** Cullen Horstmann, Kyoungtae Kim

**Affiliations:** 1 Department of Biology, Missouri State University, 901 S National, Springfield, MO 65897, USA; Horstmann95@live.missouristate.edu; 2 Jordan Valley Innovation Center, Missouri State University, 542 N Boonville, Springfield, MO 65806, USA

**Keywords:** gene expression, QDs, RNA-seq, DEGs, GFP, confocal microscopy

## Abstract

The primary focus of our research was to obtain global gene expression data in baker’s yeast exposed to sub-lethal doses of quantum dots (QDs), such as green-emitting CdSe/ZnS and InP/ZnS, to reveal novel insights on their unique mechanisms of toxicity. Despite their promising applications, their toxicity and long-lasting effects on the environment are not well understood. To assess toxicity, we conducted cell viability assays, ROS detection assays, and assessed their effects on the trafficking of Vps10-GFP toward the *trans*-Golgi network with confocal microscopy. Most notably, we used RNA-sequencing (RNA-seq) to obtain gene expression profiles and gene identities of differentially expressed genes (DEGs) in QD-treated yeast. We found CdSe/ZnS QDs significantly altered genes implicated in carboxylic acid, amino acid, nitrogen compounds, protein metabolic processes, transmembrane transport, cellular homeostasis, cell wall organization, translation, and ribosomal biogenesis. Additionally, we found InP/ZnS QDs to alter genes associated with oxidation-reduction, transmembrane transport, metal ion homeostasis, cellular component organization, translation, and protein and nitrogen compound metabolic processes. Interestingly, we observed an increase in reactive oxygen species (ROS) in CdSe/ZnS-treated cells and a decrease in ROS levels in InP/ZnS-treated cells. Nevertheless, we concluded that both QDs modestly contributed cytotoxic effects on the budding yeast.

## 1. Introduction

Quantum dots (QDs) are nano-sized semiconductor crystals well-known for their long-lasting fluorescence, photo-stability, and tunable optic properties [1,2,3]. The color of each QDs fluorescence is size-dependent, therefore, changing a QDs size will directly change its emission wavelength [3]. Soon after their development and introduction to the biological sciences, QDs proved to be powerful fluorescent probes [4,5] that work better in applications that include nucleic acid detection, protein tracking, intracellular reporting, molecular imaging, drug delivery, and tumor diagnostics [2,3,6,7]. Biological imaging with QDs is possible through modifying their surface by conjugating them with target-specific antibodies, peptides, or small molecules [2]. Despite their advantages, the use of QDs raises a lot of concern regarding their potential negative effects [1]. QD cytotoxicity has been investigated in many cell models including, but not limited to, yeast [8,9], bronchial epithelial cells [10], macrophages, lymphocytes [11], and animal models such as mice [12], rats [13], and non-human primates [14]. Some negative effects are, in part, due to their nano-size which enables them to adversely interact with organismal and microbial environments, including lung alveoli [15] and DNA within the nucleus of a cell [1,16]. To combat these potential hazards, protective semiconductor shells have been developed that coat the toxic core of QDs. Zinc sulfide (ZnS) is a protective shell, encapsulating QDs, that can be synthesized onto the core of QDs (such as CdSe and InP, respectively) and have been shown to reduce cytotoxic effects [1,17]. Despite these attempts to improve their safety, concerns regarding exposure to QDs and QD-containing products persist [1,18].

Several previous studies have revealed Cd- and Pb-based QDs to be toxic, causing significant cellular damage [1,19], but cadmium selenide, cadmium telluride, and cadmium sulfide (CdSe, CdTe, and CdS, respectively) QDs are the most widely used [20]. Cadmium is a well-known carcinogen, that has been shown to damage the liver and kidneys, and the use of Cd-QDs in electronics has been banned by the European Union [21]. According to previous studies on Cd-QDs, heavy metal ions released from the surface of degrading QDs induce toxicity by indirectly inducing the generation of reactive oxygen species (ROS) [19,22]. Due to these findings, there is still much debate over the risks of Cd-QD exposure and the continuation of their use in products and clinical research [23]. 

To overcome obstacles surrounding QD toxicity, a less toxic, Cd-free, QD could be a potential solution. A previous study provided evidence that InP/ZnS QDs are a less hazardous semiconductor nanocrystal compared to Cd-containing QDs [24]. InP/ZnS QDs, similar to CdTe-QDs, can fluoresce, which has made them a practical alternative to Cd-QDs [20]. Studies conducted in vivo found an accumulation of indium from InP/ZnS QDs remained in major organs up to 84 days after injection in BALB/c mice, but histological analysis of the organism’s blood did not reveal any discerning toxic effects [25]. A recent study revealed that InP/ZnS QDs are taken up into cells and high doses decreased cell viability and induced ROS generation and apoptosis [19].

Challenges regarding InP QDs, including their rapid oxidation and break down in biocompatible solutions, must be solved to reduce their cytotoxicity [20]. Similar to CdSe, InP QDs are coated with a ZnS-shell that improves stability and reduces toxicity [20]. Previous studies have identified a potential drawback, by demonstrating the poor coordination strength between InP-cores and ZnS-shells, which makes it difficult for the core to be fully coated, resulting in holes that expose the InP-core. The addition of a second ZnS-shell to InP/ZnS QDs has been shown to improve coverage around InP-cores and reduce cytotoxicity [20]. Additionally, they revealed InP/ZnS QDs increased ROS levels, primarily superoxide, and InP/ZnS QDs with a second ZnS-shell reduced ROS levels, in the budding yeast [20]. 

Although InP-QDs has been suggested as a potential replacement for Cd-QDs due to their similar analogous bandgap characteristics to Cd-QDs [20], we attempted to better understand the potential mechanisms of toxicity of CdSe/ZnS and InP/ZnS QDs on the budding yeast, *Saccharomyces cerevisiae*. The present study implemented high throughput technologies (RNA-seq) to assess alterations in the transcriptomic profile of QD-treated yeast and examined the cytotoxic effects in response to CdSe/ZnS and InP/ZnS exposure. Our study aims to measure toxicity by assessing each QDs effect on proliferation, ROS levels, and change in gene expression. Further, we investigated QD-mediated effects on cellular trafficking and found that CdSe/ZnS and InP/ZnS significantly alter Vps10-GFP trafficking. A detailed discussion on the potential toxic effects and their impacts on the cell caused by these quantum dots is provided in the discussion section.

## 2. Materials and Methods

### 2.1. Quantum Dots 

Green CdSe/ZnS and InP/ZnS QDs with functionalized carboxylic acid ligands were suspended in water at a concentration of 1000 μg/mL. They were obtained from NN-Labs (Fayetteville, AR, USA). CdSe- and InP-QD cores were capped with ZnS-shells to stabilize their absorption and emission wavelengths. The core/shell nanocrystal structure demonstrates brighter fluorescence and increased control over the surface chemistry. The emission wavelength of green-emitting CdSe/ZnS QDs were found to be 530–550 nm [26] and InP/ZnS QDs 530 nm +/−15 nm (NNCrystal US Corporation, Fayetteville, AR, USA) (unpublished). The size of the 530 nm CdSe/ZnS QDs were found to be 6.1–9.5 nm [26] and the 530 nm InP/ZnS QDs were found to be 3.2–4.2 nm in diameter [27]. QD size was verified using a JEOL 7900F scanning electron microscope (SEM, JOEL, Peabody, USA) with a scanning transmission electron microscopy (STEM) detector to image individual QDs, and the results were in agreement with datasheet values provided by the NNCrystal US Corporation website (nn-labs.com, January 2021) [26,27]. 

### 2.2. Growth Assay with Exposure to CdSe/ZnS and InP/ZnS QDs

*S. cerevisiae* S288C cells (MATα SUC2 gal2 mal2 mel flo1 flo8–1 hap1) were purchased from ATCC (American Type Culture Collection) (Manassas, VA, USA) and grown in synthetic-defined glucose (SD-Glu) media overnight in a shaking incubator at 30 °C. The optical density (OD) of cells was recorded at 594 nm using a BioMate 3S spectrophotometer (ThermoFisher Scientific, Waltham, MA) after being cultured for 16–18 h in a shaking incubator. Cultures were grown to 1 × 10^7^ cells/mL and inoculated in 2X SD-Glucose media to an OD of 0.01 These cells were then plated on a 96-well culture plate with CdSe/ZnS and InP/ZnS QDs at concentrations of 0, 10, 20, 50, and 100 μg/mL and 0, 1, 10, 50, and 100 μg/mL, respectively. Positive controls containing no nanomaterials and blank wells containing no cells were also incorporated. In each trial, the positive control and blank were replicated four times. Next, cells were placed on an ELx808 Absorbance Microplate Reader (BioTek, Winooski, VT, USA) and grown for 24 h at 30 °C, with the OD at 594 nm recorded every 30 min. OD readings from samples containing QDs but not cells were subtracted from each test condition, and the corrected ODs were averaged to create representative growth curves for each test concentration. The logarithmic portion of the growth curves were used to find doubling times for each QD concentration. This growth assay was carried out in triplicate.

### 2.3. Total RNA Extraction and Amplification of cDNA Libraries 

Yeast cells (S288C) were grown in SD-Glu media to mid-log phase corresponding to an OD at 600 nm of 0.3–0.6. These cells were incubated at 30 °C and shaken at 220 rpm for 5 h. This experiment was performed in triplicate consisting of three control samples (containing only SD-Glucose media and cells), three samples treated with CdSe/ZnS QDs (10 μg/mL), and three samples treated with InP/ZnS QDs (100 μg/mL). The RiboPure Yeast RNA Extraction Kit (Thermo Fisher Scientific) was used on all control and QD-treated samples. The RNA concentrations were measured with a Qubit 3.0 Fluorometer (ThermoFisher, Waltham, MA, USA) and fell within the acceptable range of 10 ng to 1 μg for library amplification. The Universal Plus mRNA-Seq Kit (NuGEN, Reddwood City, CA, USA) was used to generate adaptor-ligated sequencing-ready cDNA libraries from treated and non-treated total RNA samples. cDNA libraries were sequenced using an Illumina HiSeq 2500 Sequencing system (Kansas Medical Genome Center, Kansas City, MO, USA). One hundred nucleotides from only one end of each sequence (double-end sequencing) were completed with the cDNA libraries originated from three control, three CdSe/ZnS QD treated, and three InP/ZnS QD treated cells. 

### 2.4. Analysis of Sequencing Data

Analysis of sequencing data from cDNA sequencing was completed using usegalaxy.org (June 2020), a web-based platform for sequencing analysis. The files received from Kansas University Medical Center were uploaded to the Galaxy server and concatenated, resulting in a single file that represents each sample in a treated or non-treated group, and contained all transcript data related to those samples. A quality check was run on each file to ensure high-quality reads. The files were then groomed to be converted into Sanger format, which is required for the following steps. To ensure high fidelity, the reads were trimmed based on quality, and any bases with a quality score below 20 were removed from the reads. To eliminate the bias of primers and to ensure removal of adapters, 12 bases were trimmed from the 5′ end of the reads. The reads were then filtered, resulting in any reads shorter than 80 base pairs being removed. Reads were then aligned to the S. cerevisiae reference genome (S288C), downloaded from the Saccharomyces Genome Database (SGD) using the Tophat function on Galaxy. The transcriptome was assembled using Cufflink that uses a reference annotation, matching the reference genome. Finally, using Cuffdiff, the expression rates were found from the transcript sequences and compared between conditions, resulting in a list of DEGs. Genes with a *p*-value (q) ≤ 0.05 were considered to be statistically significant. The DEGs were then grouped into categories based on their correlating Gene Ontology terms (GO terms) acquired with the website GOrilla.com (June 2020).

### 2.5. Quantitative Reverse Transcription (RT)-qPCR

To validate our gene expression data we repeated our total RNA extraction experiment and amplified new cDNA libraries for RT-qPCR. According to our gene expression data, TIR1 and HXK1 were significantly upregulated and SPS100 and YDL012C were significantly downregulated in CdSe/ZnS and InP/ZnS-treated groups, respectively. ALG9, a housekeeping gene, was not significantly altered in either treatment group and used as the reference. DNA primers were designed and ordered for each differentially regulated gene. Next, we conducted a primer efficiency test on each primer (PowerTrack SYBR Green Master Mix, ThermoFisher Scientific, Waltham, MA, USA). Primer efficiency values fell between 1.76 and 2.14. Next, 30 ng of cDNA from each treatment group was amplified in triplicate using the PowerTrack SYBR Green Master protocol (ThermoFisher Scientific, Waltham, MA, USA). The samples were amplified in a QuantStudio 6 Pro instrument (Thermo Fisher Scientific, Waltham, MA, USA, USA). DNA amplification and the Cq values from the reference and treatment groups were averaged and used in the Pfaffl method equation to determine relative fold-changes. 

### 2.6. Measurement of Reactive Oxygen Species

Reactive oxygen species (ROS) and superoxide levels were quantified with flow cytometry in triplicate, with a total of 18 samples per experiment. Each sample was diluted to have an OD of 0.1 in 100 μL of SD-Glu media and incubated in a shaking incubator for 6 h at 30 °C. At hour 6, each sample was treated with 5 μg/mL of dihydrorhodamine 123 (DHR123) or dihydroethidium (DHE). Next, samples were incubated for another 2 h before taken to 1 mL with 1X-PBS buffer and quantified with flow cytometry (Attune NxT acoustic focusing cytometer, Life Technologies). Samples 1–9 tested for the presence of ROS by utilizing the ROS indicator DHR123. Samples 10–18 tested for the presence of superoxide with the superoxide indicator, DHE. Samples 1–3 and 10–12 had a concentration of 0 μg/mL of CdSe/ZnS and InP/ZnS QDs, respectively. Samples 4–6 and 13–15 had a concentration of 10 μg/mL CdSe/ZnS QDs and 10 μg/mL InP/ZnS QDs, respectively. Samples 7–9 and 16–18 had a concentration of 100 μg/mL CdSe/ZnS and 100 μg/mL InP/ZnS, respectively. Once measured with the Attune NxT acoustic focusing cytometer, the resulting peaks were gated and ROS quantified. The gates were customized to account for the % fluorescence from oxidized indicators (DHR123 and DHE) in their respective samples.

### 2.7. Confocal Microscopy Analysis

Wild type cells expressing Vps10-GFP were treated with CdSe/ZnS and InP/ZnS QDs, both QDs were treated at 10 μg/mL for six hours at 30 °C in a shaking incubator. After incubation with our QDs, 500 μL of each sample was transferred to a micro-centrifuge tube and centrifuged at 2000 rpm for 10 min. If a pellet was visible, we would continue and remove 300 μL of media, without disturbing the pellet. Next, we re-suspended the pellets by vortexing and transferred 2.8 μL of the concentrated sample onto a glass slide, then we gently applied a coverslip to our droplet, and visualized the cells with a confocal microscope. The green laser was used for confocal microscopy and the intensity was set to 200 to detect punctated Vps10-GFP with immunofluorescence images. We quantified the number of Vps10-GFP puncta in NTC, CdSe/ZnS- (10 μg/mL), and InP/ZnS-treated (10 μg/mL) samples. 

### 2.8. Statistical Analysis

All experiments were performed in triplicate (growth assay, total RNA extraction, ROS assay, and quantification of Vps10-GFP puncta from immunofluorescence images obtained with confocal microscopy), so each sample in every graph represents the average of three replicates. Standard deviations are represented in each bar graph by displaying error bars. GraphPad Prism 6.0 was used to conduct one-way ANOVA analysis to determine if treated-samples are statistically different from NTC samples and Dunnett’s multiple comparisons to determine a variance in treated-samples compared to NTC samples. When evaluating statistically significant differences between NTC samples and treated-samples, a *p*-value of less than or equal to 0.05 was considered statistically significant. *p*-values represent significance in our graphs using the following amount of asterisks: * *p* < 0.05, ** *p* < 0.01, *** *p* < 0.001, **** *p* < 0.0001.

## 3. Results

### 3.1. CdSe/ZnS QDs and InP/ZnS QDs Affect Normal Yeast Growth

**Figure 1:**
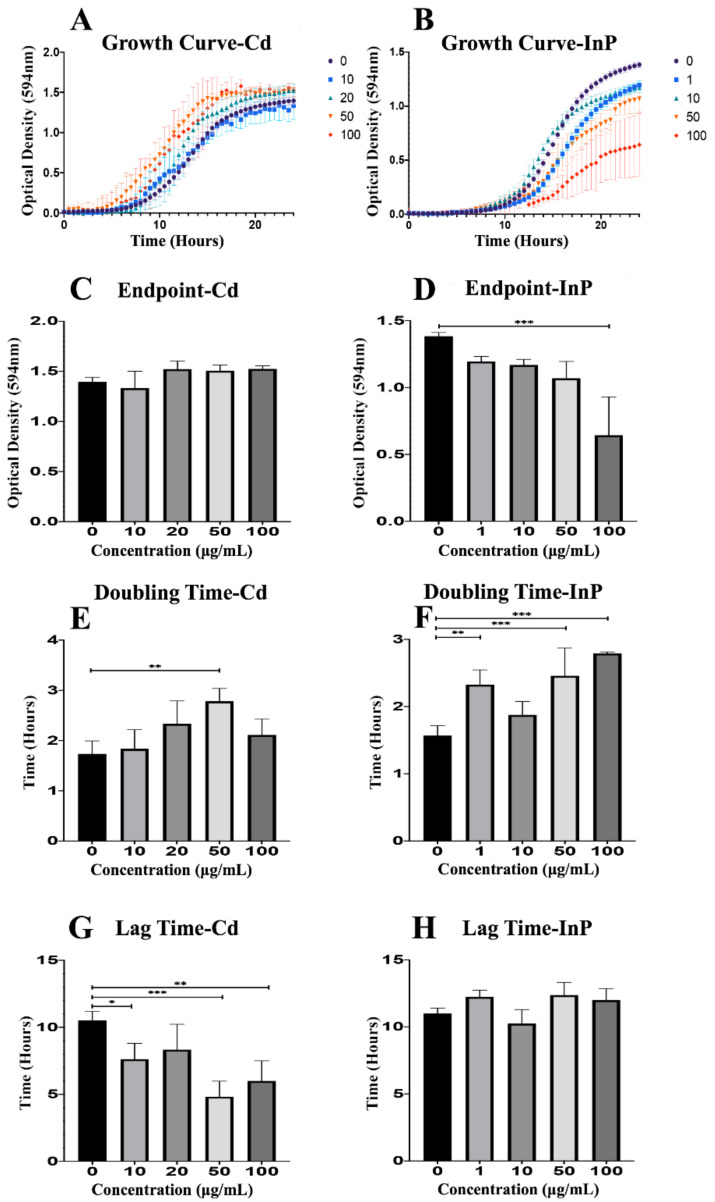
Growth assay to determine growth rates of CdSe/ZnS and InP/ZnS-treated yeast cells. (**A**,**B**) Quantification of cell optical densities over a 24-h period where the cells are treated with CdSe/ZnS and InP/ZnS quantom dots (QDs), respectively, at 30 °C while shaking. (**C**,**D**) Measurement of cell optical densities at 24 h of treatment with CdSe/ZnS and InP/ZnS QDs, respectively. The bar representing the average optical densities (ODs) (594 nm) of each concentration at the 24-h mark. (**E**,**F**) Doubling time takes place during the phase of exponential growth for the cells treated with CdSe/ZnS and InP/ZnS QDs, respectively, and was measured as the amount of time it takes for cells to double their ODs. (**G**,**H**) The mean lag time before the exponential growth phase. Significant statistical differences are represented with *(*p* < 0.05), **(*p* < 0.01), and ***(*p* < 0.001).

To fully examine the effects of CdSe/ZnS QDs on yeast growth, we recorded the optical density of each sample (0, 10, 20, 50, and 100 μg/mL CdSe/ZnS) for 24 h (Figure 1). CdSe/ZnS QDs did not have a significant effect on yeast growth curves compared to the non-treated control, but higher concentrations of CdSe/ZnS (20, 50, and 100 µg/mL) seemed to slightly stimulate growth as seen by the top 3 growth curves (red, orange, and green curves) in Figure 1A. InP/ZnS QDs significantly reduce yeast growth in a dose-dependent fashion (Figure 1B). Next, we graphed the final ODs of each sample at a steady-state on hour 24 (OD_594_ nm). We found CdSe/ZnS QDs had no significant effect on endpoints (Figure 1C) compared to the non-treated control. Once again, we show InP/ZnS QDs had a dose-dependent effect on growth. As concentrations increased, endpoints steadily decreased, and the highest concentration (100 µg/mL) was significantly reduced (Figure 1D). Furthermore, we calculated the doubling times of the yeast in each sample with the slope of the growth curve in the exponential phase (Figure 1E,F) and recorded the amount of time each sample remained in lag-phase (Figure 1G,H). Yeast treated with CdSe/ZnS QDs displayed no significant change in their endpoint OD (Figure 1C) or doubling times apart from yeast treated with 50 μg/mL CdSe/ZnS, which showed a significant increase in doubling time (Figure 1E). However, the amount of time spent in the lag-phase was significantly decreased in samples treated with 10, 50, and 100 μg/mL CdSe/ZnS (Figure 1G). The same components used to analyze the growth of yeast were investigated in samples treated with InP/ZnS QDs at concentrations of 0, 1, 10, 50, and 100 μg/mL. Interestingly, InP/ZnS QDs seemed to significantly decrease the endpoint OD at 100 μg/mL (Figure 1D) as well as significantly increase the doubling times of samples at concentrations of 1, 50, and 100 μg/mL (Figure 1F). Unlike CdSe/ZnS treated samples, yeast exposed to InP/ZnS QDs caused no significant difference in time spent in lag-phase when compared to the non-treated control (Figure 1H). Taking all the growth data obtained from CdSe/ZnS and InP/ZnS exposed yeast, a side-by-side comparison reveals each QD affects growth differently. CdSe/ZnS-treated samples significantly altered time spent in a lag phase when compared to the non-treated control and had little to no effect on the endpoint ODs or doubling times. In contrast, InP/ZnS-treated samples significantly changed the endpoint ODs and doubling times and had no significant effect on time spent in lag-phase.

Due to recent advances in high-throughput sequencing technologies, we can now identify a very broad range of genes and cellular processes that change when exposed to certain materials. All CdSe/ZnS- and InP/ZnS-treated and non-treated samples underwent a total RNA extraction followed swiftly by an mRNA purification and cDNA conversion step. Each group, performed in triplicate, was sent to the Kansas Medical Genome Center where they sequenced the cDNA in each sample with an Illumina HiSeq 2500 sequencing system (Illumina^®^, San Diego, CA, USA) that created datasets of sequenced data of each sample replicate. All cDNA datasets were returned online and then uploaded to usegalaxy.org for computational data analysis. All three control and QD-treated replicates were concatenated resulting in one joined file of the three non-treated samples, one file of the three CdSe/ZnS-treated samples, and one file of the three InP/ZnS-treated samples. Next, each cDNA dataset was checked for quality (FastQC and FASTQ Quality Trimmer, respectively). After each dataset was cleaned up, they were mapped to the *S. cerevisiae* reference genome (S288C). A combined total of 81,205,179 reads were accepted from the three non-treated controls, a total of 79,562,512 and 82,520,772 accepted reads were accepted from the three Green CdSe/ZnS-treated samples and the three InP/ZnS-treated samples, respectively. 

**Figure 2:**
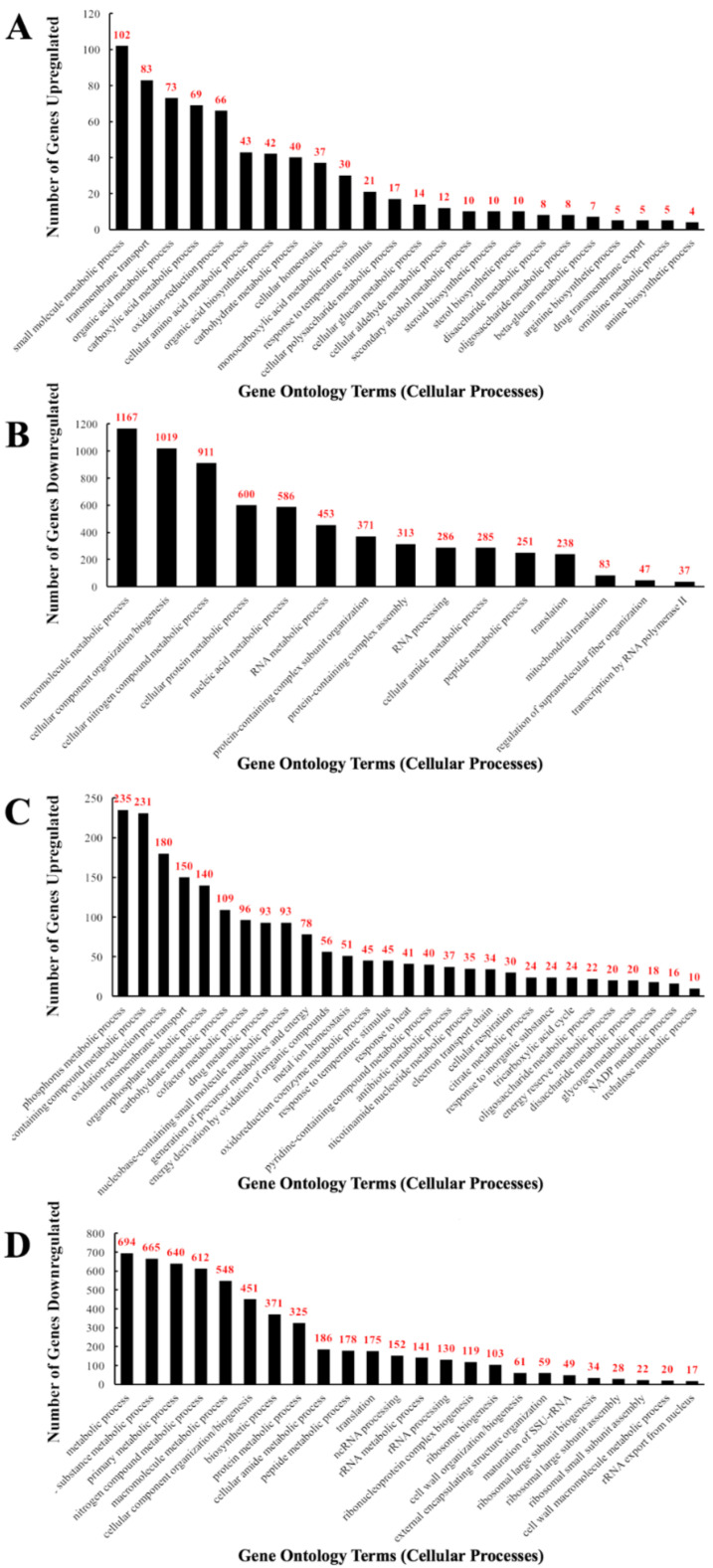
Bar graphs of the number of differentially expressed genes implicated in GO-terms that represent one specific cellular function altered when exposed to QDs in yeast. The number of differentially expressed genes (DEGs) on each bar represents the number of an altered gene for the corresponding GO-term with *p*-values of 0.05 or below. (**A**) The total number of upregulated DEGs implicated in the listed GO-terms in CdSe/ZnS-treated cells. (**B**) The total number of downregulated DEGs implicated in the listed GO-terms in CdSe/ZnS-treated cells. (**C**) The total number of upregulated DEGs implicated in the listed GO-terms in InP/ZnS-treated cells. (**D**) The total number of downregulated DEGs implicated in the listed GO-terms in InP/ZnS-treated cells.

For CdSe/ZnS QD treated samples, 606 upregulated genes were identified and found to be implicated in transmembrane transport (13.6%), carboxylic acid metabolic processes (11.4%), amino acid metabolic processes (7.1%), cellular homeostasis (6.1%), cellular glucan metabolic processes (2.3%), and drug transmembrane export (0.8%), as depicted in Figure 2A. From the GO terms listed above, 83, 69, and 37 upregulated genes were involved in transmembrane transport, carboxylic acid metabolism, and amino acid metabolism, respectively. Furthermore, 43, 14, and 5 upregulated genes were implicated in cellular homeostasis, cellular glucan metabolism, and drug transmembrane export, respectively (Figure 2A). From the pool of 2760 downregulated genes, many were involved in the macromolecule metabolic process (42.3%), component organization/biogenesis (36.9%), nitrogen compound metabolism (33.0%), protein metabolic processes (21.7%), and translation (8.6%). Among the downregulated GO terms listed above, 694, 612, 325, and 175 downregulated genes were found to be involved in metabolic processes, nitrogen compound metabolic processes, protein metabolic processes, and translation, respectively. Moreover, 152, 141, 103, 61, and 59 downregulated genes play a role in ncRNA processing, rRNA metabolic processes, ribosomal biogenesis, cell wall organization/biosynthesis, and external encapsulation structure organization, respectively. Lastly, 28, 22, and 17 downregulated genes were found to be implicated in large and small ribosomal assembly and rRNA export from the nucleus, respectively (Figure 2B). 

In InP/ZnS QD treated samples, 6488 genes were mapped to the genome and annotated, and of those genes, 2620 were found to be statistically increased or decreased compared to the non-treated controls. 1523 genes were found to be upregulated and 1097 genes downregulated. For Inp/ZnS QD treated samples, through analysis of GO terms, we have identified several upregulated cellular processes including oxidation-reduction (11.8%), transmembrane transport (9.9%), drug metabolic process (6.1%), metal ion homeostasis (3.3%), electron transport chain (2.2%), cellular respiration (1.7%), glycogen metabolic process (1.2%), and NADP metabolic process (1.1%) (Figure 2C). From our GO analysis, we determined 180, 150, 93, and 51 upregulated genes are involved in oxidation-reduction, transmembrane transport, drug metabolic processes, and metal ion homeostasis, respectively. Additionally, 34, 30, 18, and 16 upregulated genes were found to be involved in the electron transport chain, cellular respiration, glycogen metabolic processes, and NADP metabolic processes, respectively (Figure 2C). From all 1097 significantly downregulated genes, several were found to be involved in nitrogen compound metabolic processes (55.8%), cellular component organization/biogenesis (41.1%), protein metabolic processes (29.6%), translation (16.0%), ncRNA processing (13.9%), rRNA processing (11.9%), ribosome biogenesis (9.4%), cell wall organization/biogenesis (5.6%), external encapsulating structure organization (5.4%), ribosomal small and large subunit assembly (2% and 3.1%, respectively), and rRNA export from the nucleus (1.6%). GO analysis revealed that 612, 451, 325, and 175 downregulated genes play important roles in nitrogen compound metabolic processes, cellular component organization/biogenesis, protein metabolic processes, and translation, respectively. In addition, 152, 130, 103, 61, and 59 downregulated genes are involved in ncRNA processing, rRNA processing, ribosome biogenesis, cell wall organization/biogenesis, and external encapsulating structure organization, respectively. Lastly, 28, 22, and 17 downregulated genes were identified to play a role in the GO terms ribosomal small subunit assembly, ribosomal large subunit assembly, and rRNA export from the nucleus, respectively (Figure 2D).

### 3.2. RT-qPCR Validation of RNA-Seq Data 

**Figure 3:**
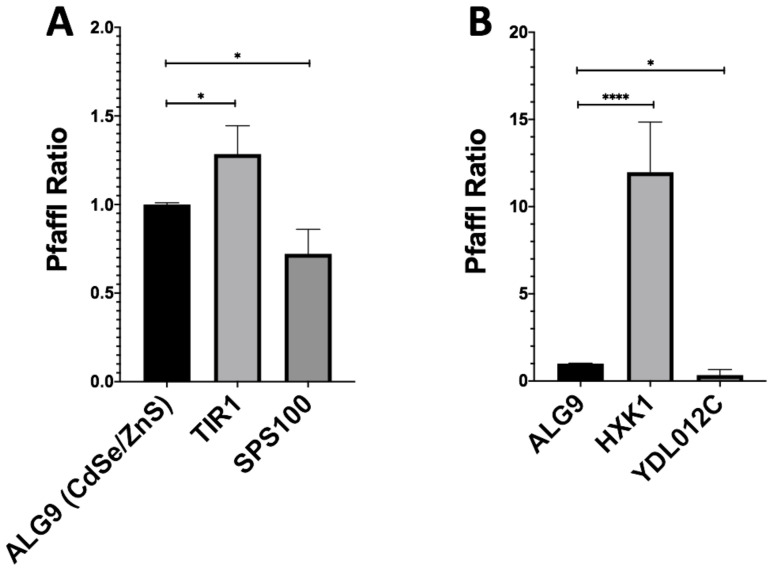
Gene expression ratios of ALG9 and one up- and downregulated gene from QD-treated samples determined by RT-qPCR. The reference gene ALG9 and other differentially expressed genes (from CdSe/ZnS and InP/ZnS-treated samples) were selected from our RNA-seq expression data and validated by determining their relative fold-change using the Pfaffl method. (**A**) Upregulated (TIR1) and downregulated (SPS100) genes with significantly altered gene expression levels Compared to ALG9 when treated with CdSe/ZnS. (**B**) Upregulated (HXK1) and downregulated (YDL012C) genes with significantly altered gene expression levels compared to ALG9 when treated with CdSe/ZnS. One asterisk (*) represents *p* < 0.05, while 4 asterisks (****) represent *p* < 0.0001.

We then wished to validate the fidelity of our RNSseq results using RTqPCR. With the Pfaffl method, we determined the fold-change of our downregulated genes (SPS100, YDL012C), upregulated genes (TIR1, HXK1), and housekeeping gene (ALG9) using RT-qPCR (Figure 3). The fold-change of CdSe/ZnS-treated SPS100 had an average fold-change of 0.72 and InP/ZnS-treated YDL012C had an average fold-change of 0.34 (Figure 3 A,B). CdSe/ZnS-treated cells displayed an elevation of TIR1 mRNA level which had an average fold-change of 1.29, and the HXK1 mRNA level in InP/ZnS-treated cells was increased more than 10 times when compared to that in non-treated cells (Figure 3 A,B). Gene expression values below 1.0 are interpreted as downregulated and expression values greater than 1.0 are interpreted as upregulated genes compared to non-treated samples. Together, our RTqPCR results clearly validated the RNAseq results.

### 3.3. ROS Quantification in Response to CdSe/ZnS and InP/ZnS Exposure

It has long been believed that the cytotoxicity of QDs is, in part, due to their ability to cause oxidative stress in cells. Most studies will measure the levels of ROS to determine how much oxidative stress QDs are inflicting in certain cells and organisms. In our study, CdSe/ZnS and InP/ZnS QDs affected yeast differently according to our growth curves and gene expression data. We decided to measure ROS levels in CdSe/ZnS and InP/ZnS treated samples to determine if one is causing more oxidative stress than the other. Additionally, Table 1 depicts multiple genes implicated in antioxidant defenses [28] and shows the effect of Cd and InP QDs on each gene’s expression to provide more insight on how they change oxidative stress response mechanisms in yeast.

**Figure 4:**
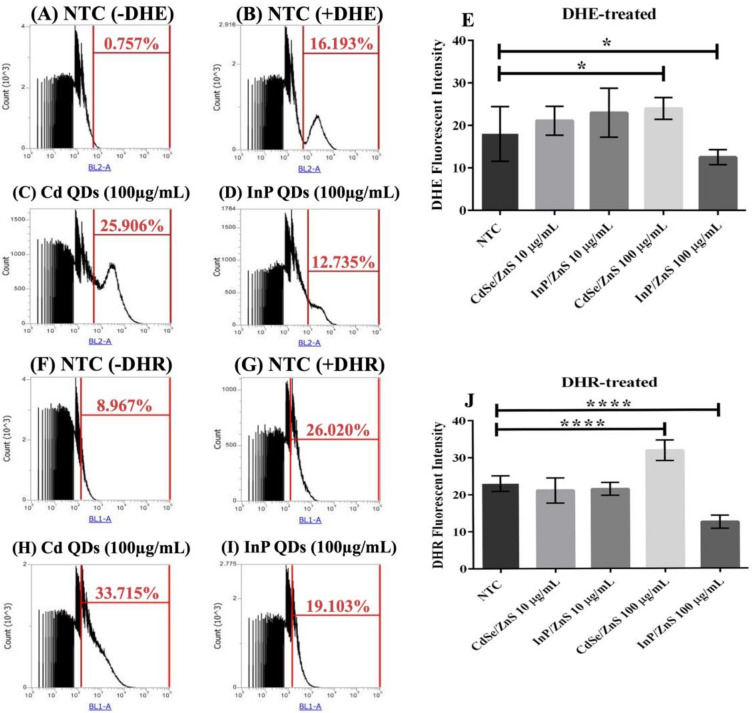
Quantification of reactive oxygen species (ROS) levels including superoxide and peroxynitrite generated by cells treated with CdSe/ZnS and InP/ZnS QDs at 10 and 100 µg/mL, respectively. Samples were treated with and incubated with dihydroethidium (DHE) or dihydrorhodamine (DHR) 2 h prior to measuring the oxidized DHE and DHR fluorescent byproducts, which we measured with flow cytometry, that allow us to observe changes in superoxide and peroxynitrite levels generated in each QD-treatment, respectively. (**A**) Non-treated sample without DHE. (**B**) Non-treated sample dyed with DHE. (**C**) DHE dyed sample treated with 100 µg/mL CdSe/ZnS. (**D**) DHE dyed sample treated with 100 µg/mL InP/ZnS. (**E**) Bar graph comparing % changes in superoxide levels. (**F**) Non-treated sample without DHR. (**G**) Non-treated sample dyed with DHR. (**H**) DHR dyed sample treated with 100 µg/mL CdSe/ZnS. (**I**) DHR dyed sample treated with 100 µg/mL InP/ZnS. (**J**) Bar graphs comparing % changes in peroxynitrite levels. One asterisk represent *p* < 0.05 and four asterisk represent *p* < 0.0001.

We quantified ROS levels (superoxide and peroxynitrite) in samples treated with CdSe/ZnS and InP/ZnS QDs at 10 and 100 µg/mL, respectively (Figure 4). The cells were cultured for six hours with the nanomaterials, then allowed to grow for 2 h with DHE (dihydroethidium) and DHR-123 (dihydrorhodamine-123) prior to measuring the oxidized DHE and DHR fluorescent byproducts, which indicate levels of superoxide and peroxynitrite, respectively (Figure 4). No significant changes in ROS levels were observed in yeast treated with 10 µg/mL CdSe/ZnS or InP/ZnS (Figure 4E,J). In cells dyed with DHE and treated with 100 µg/mL CdSe/ZnS, we observed a significant increase in superoxide levels (Figure 4C,E). In cells dyed with DHE and treated with 100 µg/mL InP/ZnS, we observed a significant decrease in superoxide levels (Figure 4D,E). In cells treated with DHR123, we observed a significant increase in peroxynitrite levels in those exposed to 100 µg/mL CdSe/ZnS QDs (Figure 4H,J) and a significant decrease in cells exposed to 100 µg/mL InP/ZnS QDs (Figure 4I,J). This suggests that both QDs have a unique effect on the generation of ROS in yeast and do not significantly affect ROS levels at concentrations lower than 10 µg/mL.

### 3.4. DEGs Implicated in Cellular Trafficking 

**Figure 5:**
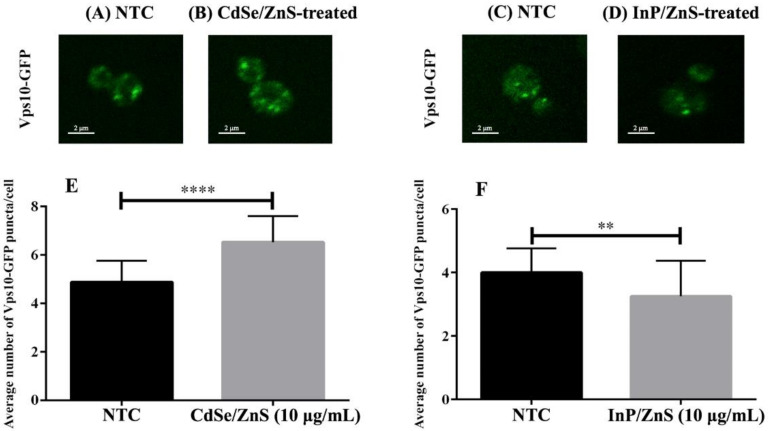
Cell images and bar graphs representing the number of Vps10-GFP puncta in CdSe/ZnS and InP/ZnS treated samples. (**A**–**D**) Representative images of wild type Vps10-GFP puncta in yeast. (**A**) Bar graph representing the average number of Vps10-GFP puncta per cell in CdSe/ZnS-treated samples. (**B**) Bar graph representing the average number of Vps10-GFP puncta per cell in InP/ZnS-treated samples. (**C**) Representative immunofluorescence image of a wild type Vps10-GFP cell from an NTC sample. (**D**) Representative immunofluorescence image of a cell from a sample treated with 10 µg/mL CdSe/ZnS. (**E**) Representative immunofluorescence image of a cell from a sample treated with 10 µg/mL InP/ZnS. (**F**) Bar graph representing the average number of Vps10-GFP puncta per cell in InP/ZnS-treated samples. Two asterisks represent *p* < 0.01 and four asterisks represent *p* < 0.0001.

To better understand the mechanisms behind CdSe/ZnS and InP/ZnS toxicity, we investigated their effects on intracellular trafficking. Vps10-GFP was used to identify alterations in the intracellular trafficking of cargo between the endosomes and Golgi. We hypothesized that CdSe/ZnS and InP/ZnS QDs would increase the number of Vps10-GFP puncta in yeast cells, more so in InP/ZnS QDs due to their more severe effects on growth. A previous study using cultured human cells revealed COOH-CdSe/ZnS QDs were continuously taken up and accumulated in the endosomes. Additionally, they noted that the QDs were internalized at a fast rate and remained persistent up to six hours, which could possibly account for the greater number and disorder of Vps10-GFP puncta in our CdSe/ZnS-treated cells, but there are very few publications on the effects of QDs on intracellular trafficking. In Table 2, we provided specific and in-depth information, such as the genes incorporated in intracellular trafficking complexes and whether those genes were upregulated or downregulated in samples exposed to CdSe/ZnS or InP/ZnS QDs. Due to the number of DEGs that we found to be involved in cellular trafficking complexes (Table 2), we decided to use confocal microscopy and yeast with GFP-tagged Vps10 (Vps10-GFP) to observe defects in trafficking. Defects in Vps10-GFP trafficking toward the trans-Golgi network were quantitated by comparing the number of Vps10-GFP puncta in QD-treated cells with untreated samples. Figure 5 revealed CdSe/ZnS QDs significantly increased the number of Vps10-GFP puncta after 6 h of incubation. In contrast, InP/ZnS QDs significantly decreased the number of Vps10-GFP puncta, resulting in fewer larger Vps10-GFP puncta compared to CdSe/ZnS.

## 4. Discussion

It has been well established that Cd-based QDs exhibit high levels of toxicity and their implementation in commercial and biomedical products has been an issue of concern. Recently, it has been suggested that Cd-based QDs could be replaced by less toxic InP-based QDs. Despite a large amount of research on Cd-QD toxicity, there have been few articles on the toxicity of InP-QDs and many researchers seemed to primarily focus on the effects QDs on growth and ROS levels. A comparison study on the toxicology of Cd- and InP-based QDs is important in determining if InP-QDs can safely replace Cd-QDs in future biological applications [24]. Regardless of which QD is more toxic, we cannot rule out the possibility that both QDs are significantly toxic, which could be possible through differences in their mechanisms of toxicity. Our study investigated the cytotoxicity of CdSe/ZnS and InP/ZnS QDs on *S. cerevisiae*, and similar to previous studies, we conducted growth and ROS assays as well. Additionally, we decided to conduct an in-depth genetic experiment (RNA-seq) to provide more specific information on cellular processes affected by analyzing changes in the quantity of mRNA transcripts in QD-treated cells. To our knowledge, the present work is the first to compare CdSe/ZnS and InP/ZnS gene expression profiles and provide representative models of their cytotoxic effects. Interestingly, we identified many DEGs implicated in endocytosis and sorting pathways. Therefore, we attempted to visualize the effects of our QDs on intracellular trafficking by treating wild-type yeast expressing Vps10-GFP with our QDs and observing any changes with confocal microscopy. Together, we observed novel differences in effects our QDs had on growth, ROS levels, and trafficking in *trans*-Golgi and transmembrane vesicle sorting pathways. 

### 4.1. Why Do InP/ZnS QDs Inhibit Proliferation?

Proliferation assays demonstrated a clear difference in effects between both QD-treatments, but InP/ZnS QDs appeared to have the greatest negative effect on growth. We showed that InP/ZnS QDs had the greatest effect on cellular growth at 100 µg/mL (Figure 1B), while CdSe/ZnS-treated cells displayed no significant difference in growth at the same concentration (Figure 1A). Our results are not consistent with a study that tested the effects of double-capped CdSe/ZnS and InP/ZnS QDs, capped with mercaptopropionic acid, on proliferation. Using a colorimetric WST-8 proliferation assay, they showed CdSe/ZnS QDs significantly reduced viability in A549 (human lung carcinoma) and SH SY5Y (human neuroblastoma) cells at concentrations as low as 10 pM, and InP/ZnS QDs had no significant effect on viability at the same concentrations [24]. We cannot be sure if These inconsistent findings could be due to a difference in surface ligands, such as the difference between carboxylated shells and shells with mercaptopropionic acid ligands. 

The MAPK pathway induces several cellular responses including proliferation, differentiation, development, inflammatory responses, and apoptosis in eukaryotic cells [29]. *MSG5*, a gene downregulated in InP/ZnS-treated samples, encodes a nuclear and cytoplasmic protein that inhibits the MAPK pathway through regulating MAPK nuclear export. One possible explanation for the reduction in proliferation with InP QDs (Figure 1B) could be due to an *MSG5*-mediated immune response, triggering apoptosis. Apoptosis occurs normally in eukaryotic cells, for instance, in development, aging, and maintaining homeostasis in cell populations and is an important immune defense mechanism that occurs when cells become damaged by disease and noxious agents [30]. It is important to note that many conditions/stimuli can trigger apoptosis, and activation of apoptosis can differ between cell-types [30]. We first suspected that the greater impact on proliferation, in InP/ZnS-treated cells, was caused by a QD-mediated elevation in ROS, but after measuring ROS levels in QD-treated cells, our results suggested otherwise. A recent study that investigated the toxicity of the exact same InP/ZnS QDs on HeLa cells, found QD-treatments, at both 69 and 167 µg/mL, induced late apoptosis 4417% more than the non-treated controls [27]. This suggests that the observed reduction in proliferation by InP QDs might be primarily due to the activation of apoptosis pathways instead of increased ROS levels in InP/ZnS-treated cells. 

### 4.2. CdSe/ZnS and InP/ZnS QDs Have Opposite Effects on ROS Generation

We found CdSe/ZnS QD treatments (100 µg/mL) significantly increased the levels of ROS, including superoxide and Peroxynitrite, and InP/ZnS (100 µg/mL) statistically significantly decreased both ROS. Interestingly, a recent study that measured ROS levels in InP/ZnS-treated (69 and 167 µg/mL InP/ZnS) HeLa cervical cancer cells found InP/ZnS exposure to significantly decrease superoxide levels and significantly increase peroxynitrite levels. The decrease in superoxide levels they reported is consistent with our findings, but the increased peroxynitrite levels in their experiments contradict the effects of InP/ZnS on peroxynitrite levels in yeast. Interestingly, in a 2019 study, we investigated the effects of yellow CdSe/ZnS QDs on superoxide levels in yeast and found no significant change in ROS levels, but cells were treated with a much lower concentration (20 µg/mL) than in the current study (up to 100 µg/mL) [8]. Additionally, a study on the cytotoxicity of InP/ZnS QDs used spin-trap electron paramagnetic resonance spectroscopy and reporter assays that revealed InP/ZnS considerably increased superoxide and hydroxyl radical levels in many cell types (NIH3T3 fibroblasts, KB cells, B16 murine melanoma cells, and MDA-MB-231 breast adenocarcinoma cells) [20]. These types of discretions in ROS production are not uncommon amongst different cell-types, including closely related, eukaryotic, microorganisms [27].

**Figure 6:**
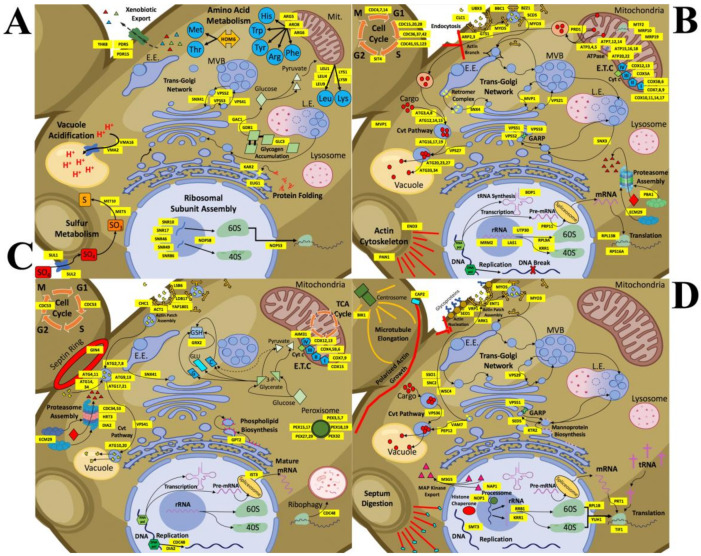
Detailed schematic models representing changes in cellular processes with CdSe/ZnS QDs or InP/ZnS QDs in the budding yeast. Cellular processes are illustrated and labeled with the genes involved and whose normal regulation has been up or downregulated. (**A**) CdSe/ZnS QD exposure causes an increase in the expression of genes involved in cellular processes such as transmembrane transport/cellular homeostasis, vacuole acidification, amino acid metabolic activity, protein folding, and trafficking within the *trans*-Golgi network. (**B**) Decreased expression in many genes treated with CdSe/ZnS has revealed many important downregulated processes involved in endocytosis, the Cvt pathway, rRNA processing, proteasome assembly, metabolic activity, cell cycle regulation, and trafficking within the *trans*-Golgi network. (**C**) Yeast exposed to InP/ZnS QDs has upregulated many genes required for processes like endocytosis, peroxisome assembly, proteasome assembly and activity, the Cvt pathway, the ETC, and oxido-reduction metabolic processes. (**D**) Several more processes are downregulated when exposed to InP/ZnS QDs including endocytosis, rRNA processing, translation, the Cvt pathway, actin and microtubule growth, vesicle fusion, and protein targeting to the plasma membrane, and some *trans*-Golgi network trafficking.

Our RNA-seq analysis identified many mitochondrial genes, involved in ATPase activity, ETC, protein degradation, and mitochondrial translation, which were significantly downregulated in the presence of CdSe/ZnS QDs (Figure 6B). We conjecture that the significant decrease in metabolic activity, seen in CdSe/ZnS-treated samples, is directly associated with an increase in ROS levels. We observed considerably fewer downregulated mitochondrial genes in InP/ZnS-treated samples (Figure 6D) compared to CdSe/ZnS-treated samples (Figure 6B). On the other hand, our gene data revealed our InP/ZnS-treated cells significantly increased the expression of genes implicated in antioxidant defense activity, such as *SOD2*, *CTT1*, *GPX1*, *COQ3*, *APN1*, *GRX2*, *GLR1*, *TRR2*, *ZWF1,* and *UBI4* (Table 1). Additionally, we found an increase in the transcription of genes implicated in peroxisome structure and activity (*PEX3*, *PEX5*, *PEX7*, *PEX15*, *PEX17*, *PEX18*, *PEX19*, *PEX27*, *PEX29*, and *PEX32*), which are organelles that play a role in metabolism, signaling, and ROS detoxification. The different ROS levels and gene expression profiles of CdSe/ZnS and InP/ZnS-treated cells could be a consequence of their unique physiochemical characteristics that affect their uptake and trafficking [31]. However, results on QD toxicity can differ drastically due to different methods of synthesizing QDs, and it is not uncommon for QDs from one batch to affect organisms differently than the same QDs from a separate batch [20]. Future studies should characterize the cellular uptake mechanisms used by each type of QD, the rate of their degradation, and their intracellular trafficking to better understand the associated cellular interactions and mechanisms of toxicity.

### 4.3. Comparing Gene Expression Profiles of Yeast Exposed to CdSe/ZnS and InP/ZnS QDs

We provide a model that depicts specific upregulated genes and potential physiological changes induced by exposure to CdSe/ZnS QDs (Figure 6A). Of the upregulated genes, the most serious seems to be those involved in transmembrane transport/cellular homeostasis, vacuole acidification, amino acid metabolic activity, protein folding (Table 3), and cellular trafficking (Table 2). In addition, several genes implicated in pre-RNA processing of ribosomal subunits (*SNR10*, *SNR17*, *SNR46*, *SNR49*, *SNR86*, and *NOP58*), protein folding (*KAR2* and *EUG1*), and amino acid metabolism (*ARG5*, *ARG6*, *ARO8*, *LYS1*, *LYS9*, *LEU1*, *LEU4*, and *LEU9*) were significantly upregulated. The upregulation of many genes implicated in protein production could be in response to a high level of nonfunctional proteins that might have been damaged by significantly increased superoxide levels [32,33] resulting from CdSe/ZnS exposure or CdSe/ZnS-protein interactions within the cells. We also noticed two upregulated genes, *YDR5* and *YDR15*, which are both ATP-binding cassette (ABC) transporters implicated in yeast’s drug efflux [34,35]. It is generally believed that these ABC transporters play an important role in cellular detoxification and their upregulation suggests that yeast cells are interpreting the presence of CdSe/ZnS QDs as a xenobiotic attack [34]. The upregulation of genes involved in cellular detoxification could explain why CdSe/ZnS had no significant effect on cellular growth (Figure 1A). However, we still do not fully understand the mechanisms involved in shortening the lag phase of samples exposed to CdSe/ZnS (Figure 1G).

The expression of genes implicated in endocytosis (*ARP2*, *ARP3*, *GTS1*, *CLC1*, *BBC1*, *BZZ1*, *SCD5*, *MYO3*, and *MYO5*) and the ETC function (*COX5A*, *COX5B*, *COX6*, *COX7*, *COX8*, *COX9*, *COX10*, *COX11*, *COX12*, *COX14*, and *COX17*) was significantly lowered in CdSe/ZnS-treated samples (Figure 6B) [31,34,35]. Additionally, genes implicated in proteasome assembly (*ECM29* and *PBA1*) were downregulated, suggesting an unbalanced protein degradation and production under unfavorable conditions or unbalanced ROS levels with these QDs. This idea is consistent with the prior finding where the function of 26S proteasomes was crippled by the oxidative stress caused by H2O2 [36]. Interestingly, we found the expression of genes implicated in *trans*-Golgi network trafficking (*SNX3*, *SNX4*, *MVP1*, *Vps21*, *Vps51*, *Vps52*, and *Vps53* implicated in transport between the endosome and Golgi) was downregulated in CdSe/ZnS-treated cells and after further testing, we observed a defect in trafficking through an increased number of Vps10-GFP puncta (Table 4) [31]. 

**Figure 7:**
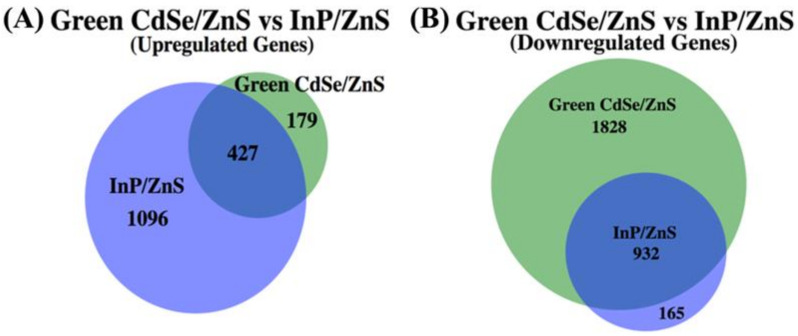
A proportionately accurate Venn-diagram that represents the number of up- and downregulated genes whose expression has been significantly changed when exposed to CdSe/ZnS QDs or InP/ZnS QDs. The overlapped segments of each Venn-diagram represent genes that are differentially expressed when exposed to CdSe/ZnS and InP/ZnS QDs. The portions of the Venn-diagram that do not overlap are up- or downregulated genes that become differentially expressed only when exposed to CdSe/ZnS QDs or InP/ZnS QDs. (**A**) Every gene whose expression was significantly increased when compared to the normal gene expression in yeast cells when exposed to CdSe/ZnS or InP/ZnS QDs. (**B**) Every gene whose expression was significantly decreased when exposed to CdSe/ZnS or InP/ZnS QDs.

We also created a detailed model of the potential effects induced by InP/ZnS QDs (Figure 6C). InP/ZnS exposure upregulates approximately 3 times as many genes compared to CdSe/ZnS-treated samples (Figure 7A). Significantly upregulated processes in InP/ZnS-treated samples include endocytosis, peroxisome assembly, proteasome assembly/activity, the Cvt pathway, the ETC, and oxide reduction metabolic processes (Table 3). Interestingly, we found the downregulation of ETC genes to be unique to CdSe/ZnS-treated cells. An opposite effect on ETC was observed in InP/ZnS-treated samples where ETC genes were significantly upregulated, including *AIM31*, *COX4*, *COX5B*, *COX6*, *COX7*, *COX9*, *COX12*, *COX13*, and *COX15* (Figure 6C). We also found many genes associated with endocytosis to be downregulated in CdSe/ZnS-treated samples, but significantly up- and downregulated in InP/ZnS-treated samples. Upregulated endocytosis genes included *LSB6*, *SDB17*, *YAP1801*, *CHC1*, and *ACT1*, while downregulated endocytosis genes consisted of MYO3, *MYO5*, *ENT1*, *ARK1*, and *VRP1* (Table 5). Interestingly, our gene expression analysis found proteasome assembly to be a downregulated process in CdSe/ZnS-treated samples (*PBA1* and *ECM29*) and upregulated in InP/ZnS-treated samples (*ECM29*, *CDC34, CDC53*, *HRT3*, and *DIA2*). 

InP/ZnS significantly downregulates approximately half the number of genes when compared to the number of downregulated genes from CdSe/ZnS-treated cells (Figure 7B). DEGs that are depicted in Figure 5D are significantly downregulated processes including endocytosis, rRNA processing, translation, the Cvt pathway, actin and microtubule growth, vesicle fusion, and protein targeting to the plasma membrane, and *trans*-Golgi network trafficking (Table 6). A gene encoding a microtubule plus-end tracking protein, *BIK1*, was downregulated in InP/ZnS-treated samples. These proteins are known to play roles in key cellular processes including cell motility, intracellular trafficking, and pathways that align the mitotic spindle with the division axis of the cell, making them crucial in cell division [37]. Another important player implicated in cell division is a gene encoding a capping protein, *CAP2*. In samples exposed to InP/ZnS, *CAP2* was significantly downregulated. In yeast, single actin monomers are used to synthesize actin cables involved in polarized growth, and in yeast lacking *CAP1* or *CAP2* have low levels of free actin due to excessive F-actin assembly in cortical patches, which is detrimental to the assembly on actin cables [38]. These genes, downregulated in InP/ZnS-treated samples, might contribute to InP/ZnS QDs’ negative impact on proliferation compared to CdSe/ZnS QDs. Additionally, we found noticeably fewer DEGs involved in endocytosis, SNAREs, and the Retromer when treated with InP/ZnS (Table 2), which suggests different uptake and trafficking mechanisms are at work compared to CdSe/ZnS-treated samples. 

### 4.4. Effects of CdSe/ZnS and InP/ZnS QDs on the Intracellular Trafficking of Vps10-GFP

Cargo trafficking in yeast is primarily done by a complex endosomal system that sorts lipids, proteins, and a variety of cargo. The system begins when cargo is endocytosed and trafficked to the early endosome where it is sorted and either sent to the lysosome for degradation or targeted to the Golgi or plasma membrane through a retrograde or recycling pathway. Endosome-to-Golgi retrograde and Endosome-to-plasma membrane recycling pathways orchestrate the re-use of sorting receptors and are important in creating the lysosome and determines what is sent to compose the plasma membrane. These pathways are responsible for many cellular functions including homeostasis and quality control of lipids and proteins. Negatively impacting the endosomal sorting pathways have been linked to many diseases including, but not limited to, cancer, Parkinson’s disease, and Alzheimer’s [39].

Vps10 is an integral sorting receptor protein that plays a role in the sorting of newly synthesized carboxypeptidase A at the *trans*-Golgi and that shuttles back and forth between the *trans*-Golgi and the endosome [39]. We treated yeast containing Vps10-GFP with our QDs and observed alterations in the phenotypes compared to non-treated cells via confocal microscopy. [31]. We found significant differences in the number of puncta in QD-treated cells. CdSe/ZnS QDs significantly increased the number of puncta and InP/ZnS QDs significantly decreased the number in yeast. [31], both of our QDs have the same carboxylic acid ligands on their surface. Additionally, each QD possesses identical ZnS shells that surround their respective cores (CdSe and InP). This suggests that differences in Vps10-GFP phenotypes are due to their different physiochemical effects of CdSe and InP-cores. It is important to note that understanding the cellular mechanisms that are coordinated by their different physiochemical properties is essential in evaluating QD toxicity and is still poorly understood.

### 4.5. Comparing the Biological Effects of CdSe/ZnS with Known Biological Effects of Cd in Saccharomyces Cerevisiae

Many types of metals and metalloids are widespread in nature and can accumulate to high concentrations locally. These metals are not QDs but greatly affect biological systems. Organisms have dealt with metals and evolved proteins that require metals for catalytic functions and maintaining the correct structure [40]. On the other hand, some metals such as Cd interact and inhibit enzymes that disrupt normal cellular processes and contribute to their toxicity, however, relatively little is known about their molecular mechanisms of toxicity. 

Using transcriptomic experiments and bioinformatics analysis, common metal-responsive (CMR) genes have been identified. The transcriptome changes made when exposed to most metals were analyzed and similar changes in gene regulation were observed, and make up the CMR genes [40]. CMR genes were enriched with GO-terms of biological processes including metal ion transport/homeostasis, ROS detoxification, carbohydrate metabolism, fatty acid metabolism, and RNA polymerase II transcription [40]. Our GO-term analysis of genes differentially expressed by CdSe/ZnS QDs were not enriched with similar GO-terms, which suggests that their transcriptomic profile is not similar to that of yeast exposed to Cd. Interestingly, the transcriptomic profile of cells treated with InP/ZnS had many similar GO-terms to the CMR genes, which could suggest InP/ZnS QDs affect yeast very similarly to toxic metals and metalloids. 

Cd metals specifically inhibit proteins that protect cells from oxidative stress including glutathione and thioredoxins. When these enzymes are inhibited it results in an increase of ROS levels due to a decrease in functional antioxidant genes [40]. Our RNA-seq data revealed that the CdSe/ZnS-treated group significantly downregulated two out of three thioredoxins (TRX1 and TRX2) Alternatively, the InP/ZnS-treated group did not significantly change the expression of any thioredoxin. These results suggest that CdSe/ZnS QDs and Cd metal have different mechanisms of toxicity on yeast. 

## 5. Conclusions

In the present study, we provided evidence that CdSe/ZnS and InP/ZnS QDs have a mild cytotoxic effect on yeast. In comparison, each QD appeared to exert unique cytotoxic effects in a variety of tests. CdSe/ZnS QDs differentially regulated gene expression in a greater number of genes, including all up and downregulated genes, than InP/ZnS QDs. Notably, InP/ZnS QDs upregulated hundreds of more genes than CdSe/ZnS QDs. Interestingly, with another cytotoxic experiment, we observed InP/ZnS QDs had a greater and dose-dependent effect on proliferation whereas we observed no significant change in CdSe/ZnS-treated samples. As depicted in our working models, we show a significant downregulation in mitochondrial/ETC function and genes implicated in *trans*-Golgi network trafficking in the presence of CdSe/ZnS QDs more so than in InP/ZnS-treated samples. Additionally, our confocal microscopy analysis revealed changes in Vps10-GFP trafficking in both QDs. It is generally believed that QDs, such as CdSe/ZnS and InP/ZnS, are trafficked and processed differently due to their individual physio-chemical properties. Based on our results, each QD induced dissimilar cytotoxic effects in our experiments on the budding yeast. Currently, we understand little about the physiochemical effects, and the mechanisms behind QD toxicity must be solved before they can be used in the life sciences or medicine. It is essential, to our health and the environment, that the toxicity of all QDs used, industrially or commercially, are thoroughly tested and understood in diverse cell types and organismal models. The next steps in studying QD toxicity should include proteomic analysis to identify novel protein interactions and better understand the effects on how their physiochemical properties coordinate their toxicity.

## Figures and Tables

**Table 1:** A table of genes implicated in antioxidant defense. It represents upregulated and downregulated genes in yeast exposed to CdSe/ZnS and InP/ZnS QDs. All genes incorporated in this table had *p*-values of 0.05 or below.

Yeast Antioxidant Genes	Function	Cd Up-Reg.	Cd Down-Reg.	InP Up-Reg.	InP Down-Reg.
Primary Antioxidant Defenses					
SOD1 (cytoplasmic superoxide dismutase)	Dis-mutation of superoxide radicals	x	*SOD1*	x	x
SOD2 (mitochondrial superoxide dismutase)		x	x	*SOD2*	x
CTT1 (cytoplasmic catalase T)	Decomposition of hydrogen peroxide	x	x	*CTT1*	x
GPX1–GPX3 (glutathione peroxidases)	Reduction of hydrogen peroxide, Reduction of alkyl hydro-peroxides	x	*GPX1*, *GPX2*	*GPX1*	*GPX2*
TRX2 (cytoplasmic thioredoxin)	Reduction of hydrogen peroxide and alkyl hydro-peroxides	x	*TRX2*	x	x
TRX3 (mitochondrial thioredoxin)		x	*TRX3*	x	x
COQ3 (ubiquinol)	Scavenging of perferryl, lipid, and lipid peroxyl radicals	x	x	*COQ3*	x
Table					
OGG1 (8-oxoguanine glycosylase/lyases)	Excision of oxidized DNA bases	x	*OGG1*	x	x
APN1 (AP endonuclease)	Cleavage of apurinic/apyrimidinic (AP) sites, Generation of 3′-hydroxyl groups at AP sites	x	*APN1*	*APN1*	x
GSH1 (glutathione)	Reduction of protein disulfides	*GSH1*	x	x	x
GRX2 (glutaredoxin)	Reduction of disulfides	x	x	*GRX2*	x
TRX2 (thioredoxin)	Reduction of protein disulfides, Reduction of oxidized glutathione	*TRX2*	x	x	x
GLR1 (glutathione reductase)	Reduction of oxidized glutathione	*GLR1*	x	*GLR1*	x
TRR2 (mitochondrial thioredoxin reductase)	Reduction of oxidized thioredoxin	x	x	*TRR2*	x
ZWF (glucose-6-phosphate dehydrogenase)	Reduction of NADP+ to NADPH	x	x	*ZWF1*	x
UBI4 (polyubiquitin)	Tagging oxidized proteins for degradation by the 26S proteasome	*UBI4*	x	*UBI4*	x

**Table 2:** A table that compares upregulated DEGs involved in cellular trafficking processes when exposed to green CdSe/ZnS and InP/ZnS QDs. The processes of interest include SNARE components, the Retromer, Sorting Nexin, and GARP complexes. All genes incorporated in this table had P-values of 0.05 or below.

Complex	CdSe/ZnS-Treated	InP/ZnS-Treated
Upregulated Genes		
SNARE	x	*NYV1, TLG2, SPO20, YPT7*
Retromer	x	x
Sorting Nexin	*SNX41*	*SNX4, SNX41*
GARP	x	*VPS52, VPS54*
Downregulated genes		
SNARE	*PEP12, VTI1, NYV1, YKT6, VAM3, VAM7, TLG1, TLG2, SSO1, SSO2, SEC9, SNC1, SNC2, SED5, GOS1, UFE1, USE1, SEC22, BOS1, BET1, VPS21*	*PEP12, VAM7, SSO1, SNC2, SED5*
Retromer	*VPS5, VPS17. VPS29, VPS35*	*VPS29*
Sorting Nexin	*SNX3, SNX4, MVP1*	x
GARP	*VPS51, VPS52, VPS53*	*VPS51*

**Table 3:** A table listing the GO-terms and genes in Figure 6A. It represents the multiple upregulated cellular processes and genes in yeast exposed to 10 µg/mL CdSe/ZnS QDs. All genes incorporated in this table exhibited expression levels significantly higher than expression levels of NTC samples.

Upregulated GO-Terms (CdSe/ZnS)	Genes in Figure 6A
Transmembrane transport/cellular homeostasis	*YHK8, PDR5,* and *PDR15*
Vacuole acidification	*VMA2* and *VMA16*
Amino acid metabolic activity	*ARG5, ARO8, ARG6, LEU1, LEU4, LEU9, and LYS1/9*
Protein folding	*KAR2* and *EUG1*
Trafficking within the *trans*-Golgi network	*VPS41 and SNX41*

**Table 4:** A table listing the GO-terms and genes in Figure 6B. It represents the multiple downregulated cellular processes and genes in yeast exposed to 10 µg/mL CdSe/ZnS QDs. All genes incorporated in this table exhibited expression levels significantly lower than expression levels of NTC samples.

Downregulated GO-Terms (CdSe/ZnS)-Downregulated)	Genes in Figure 6B
Endocytosis	*MYO3, MYO5, CLC1, ARP2, ARP3, BZZ1, SCD5, BBC1,* and *GTS1*
The Cvt pathway	*ATG3, ATG4, ATG8, ATG12, ATG14, ATG15, ATG16, ATG17, ATG19, ATG20, ATG23, ATG27, ATG33, and ATG34*
rRNA processing	*MRM2, LAS1, UTP30, RPL9A, and KRR1*
Proteasome assembly	*PBA1* and *ECM29*
Metabolic activity	*ATP3, ATP4, ATP5, ATP7, ATP12, ATP14, ATP15, ATP16, ATP18, ATP20, ATP22, MRP10, MRP19, and MTF2*
Cell cycle regulation	*CDC4, CDC7, CDC14, CDC15, CDC20, CDC28, CDC36, CDC37, CDC42, CDC45, CDC55, CDC123, and SIT4*
Cellular trafficking	*SNX3, SNX4, MVP1, VPS21, VPS51, VPS52, and VPS53*

**Table 5:** A table listing the GO-terms and genes in Figure 6C. It represents the multiple upregulated cellular processes and genes in yeast exposed to 100 µg/mL InP/ZnS QDs. All genes incorporated in this table exhibited expression levels significantly higher than expression levels of NTC samples.

Upregulated GO-Terms (InP/ZnS)	Genes in Figure 6C
Endocytosis	*LSB6, SDB17, YAP1801, CHC1, and ACT1*
Peroxisome assembly	*PEX3, PEX5, PEX7, PEX15, PEX17, PEX18, PEX19, PEX27, PEX29, and PEX32*
Proteasome assembly and activity	*ECM29, CDC34/53, HRT3, and DIA2*
The Cvt pathway	*ATG2, ATG4, ATG7, ATG8, ATG9, ATG10, ATG11, ATG13, ATG14, ATG17, ATG20, ATG21, ATG34, and VPS41*
ETC	*AIM31 and COX4, COX5B, COX6, COX7, COX9, COX12, COX13, and COX15*
Oxido-reduction metabolic processes	*GRX2*

**Table 6:** A table listing the GO-terms and genes in Figure 6D. It represents the multiple downregulated cellular processes and genes in yeast exposed to 100 µg/mL InP/ZnS QDs. All genes incorporated in this table exhibited expression levels significantly lower than expression levels of NTC samples.

Downregulated GO-Terms (InP/ZnS)	Genes in Figure 6D
Endocytosis	*MYO3, MYO5, ENT1, ARK1, and VRP1*
rRNA processing	*RRB1, KRR1, RPL1B, and YUH1*
Translation	*PRT1 and TIF1*
The Cvt pathway	*VPS36*
Actin and microtubule growth	*CAP2 and BIK1, respectively*
Vesicle fusion and protein targeting to the plasma membrane	*SSO1, SNC2, and WSC4, respectively*
Cellular trafficking	*SED5, VAM7, PEP12, VPS29, and VPS51*

## Data Availability

Our data is available and publicly archived at https://www.ncbi.nlm.nih.gov/geo/query/acc.cgi?acc=GSE165805 (15 January 2021).
